# Three new species of *Eucorydia* Hebard, 1929 (Blattodea, Corydioidea, Corydiidae) from China, with notes on female genitalia

**DOI:** 10.3897/zookeys.1293.199475

**Published:** 2026-09-28

**Authors:** Ting-Ting Cai, Wei Han, Zong-Qing Wang, Yan-Li Che

**Affiliations:** 1 College of Plant Protection, Southwest University, Beibei, Chongqing 400715, China College of Plant Protection, Southwest University Beibei China; 2 Key Laboratory of Agricultural Biosafety and Green Production of Upper Yangtze River (Ministry of Education), Southwest University, Chongqing 400715, China Key Laboratory of Agricultural Biosafety and Green Production of Upper Yangtze River (Ministry of Education), Southwest University Chongqing China

**Keywords:** ASAP, cockroach, DNA barcoding, GMYC

## Abstract

Three new species of *Eucorydia* from China are described using an integrative taxonomic approach combining morphology and *COI* sequences, with ASAP and GMYC methods: *Eucorydia parva* Cai & Che, **sp. nov**., *Eucorydia qinlingensis* Cai & Che, **sp. nov**., and *Eucorydia absens* Cai & Che, **sp. nov**. The female genitalia are illustrated and comparatively analyzed, demonstrating their taxonomic significance. Although genetic divergence between the zonata population of *E. dasytoides* and *E. qinlingensis* is low, phylogenetic analysis supports the distinctiveness of the three new species. A key to the Chinese species of *Eucorydia* is also provided.

## Introduction

*Eucorydia* Hebard, 1929 exhibits striking metallic coloration and bright markings, making it one of the most morphologically and aesthetically distinct lineages within Corydioidea. The genus is widely distributed across East Asia, Southeast Asia, and South Asia, with 22 known species worldwide, ten of which occur in China (Asahina 1971; Woo et al. 1986; Qiu et al. 2017; Yanagisawa et al. 2021a).

Early taxonomic studies relied primarily on external morphological characters, such as body color, tegminal and abdominal markings (Princis 1963; Asahina 1971; Woo et al. 1986; Woo and Feng 1988). Subsequent revisions incorporated male genitalia, greatly improving species delimitation (Qiu et al. 2017, 2019). More recently, molecular approaches, including genetic distance analyses and phylogenetic inference, have further enhanced taxonomic resolution within the genus (Yanagisawa et al. 2021a, 2021b, 2021c). Despite these advances, studies of *Eucorydia* remain strongly biased toward males. Females have received little attention, with most studies limited to simple descriptions and comparisons of external morphology (Qiu et al. 2017). However, previous research has shown that the sclerotized structures of female genitalia in Blattodea are stable and species-specific, serving as important diagnostic characters (McKittrick 1964; Zhu et al. 2022; Valero-Pérez 2023; Han et al. 2024). Such characters have not yet been systematically investigated in *Eucorydia*. Furthermore, molecular data for Chinese species remain limited, despite China harboring nearly half of the known diversity of the genus.

Cytochrome c oxidase subunit I (*COI*), especially the standard 658-bp barcode fragment, is widely recognized as a rapid and reliable molecular marker for species identification (Hebert et al. 2003; Liao et al. 2019). It has been successfully applied to insect species delimitation using the Assemble Species by Automatic Partitioning (ASAP) and Generalized Mixed Yule Coalescent (GMYC) methods (Bai et al. 2018; Puillandre et al. 2021; Neo et al. 2025).

In this study, we re-evaluate the species diversity of *Eucorydia* in China using an integrative approach combining morphology and molecular data. Based on detailed morphological comparisons and phylogenetic analysis of *COI* sequences, together with ASAP and GMYC, we describe three new species within this genus. Furthermore, we provide the first comparative analysis of female genitalia in this genus and evaluate their taxonomic significance.

## Materials and methods

### Material

All specimens examined in this study, including the type specimens, are deposited in the College of Plant Protection, Southwest University, Chongqing, China (**SWU**). A total of 13 female specimens were examined, including *E. westwoodi* (2), *E. dasytoides* (7), *E. linglong* (1), *E. guilinensis* (1), and *E. qinlingensis* (2).

For genitalia dissection, the terminal 3–4 abdominal segments were excised, soaked in 10% NaOH, and heated for 10–20 min to digest soft tissues. DNA extraction, PCR amplification, and sequencing followed Han et al. (2022). Observations were conducted in glycerol using a SZ780 stereomicroscope. Images were captured using a Leica M205A stereomicroscope and processed with Adobe Photoshop 2024.

Terminology for external morphology follows Roth (2003); male genitalia follows Klass (1997) and Qiu et al. (2017); female genitalia follows McKittrick (1964). The term of meristal annulus follows Fujita and Machida (2014).

### Abbreviations in this paper are as follows

**L1, L2, L3, L4M, L4N** sclerites of the left complex

**Sc** subcosta

**TX** supra-anal plate

**SVII** subgenital plate

**pp**. paraprocts

**v.I** first valve

**v.II** second valve

**v.III** third valve

**cp**. crosspiece

**T** tergite

**R1M, R2, R3, L7** sclerites of the right phallomere and appendage sclerite

**RA** radius anterior

**p.l**. posterior lobes of valvifer II

**ltst. IX** laterosternite IX

**vlf.I** first valvifer

**pt**. paratergites

**sp.pl**. spermathecal plate

**bsv**. basivalvula

**vst.s** vestibular sclerite

**S** sternite

### Sequence processing and phylogenetic analyses

A total of 23 *COI* sequences (Table 1) were included, of which 13 were newly generated and 10 were obtained from GenBank. *Ergaula nepalensis* Saussure, 1893 and *Diploptera punctata* (Eschscholtz, 1822) were used as outgroup. All 13 newly generated sequences were submitted to GenBank with accession numbers of PZ945043–PZ945055. The *COI* fragments were aligned using the MUSCLE algorithm in MEGA 11 (Tamura et al. 2021). Interspecific and intraspecific genetic distances were calculated using the Kimura 2-parameter (K2P) model (Kimura 1980).

**Table 1.** Samples used in the phylogenetic analysis.

**Species**	**Abbreviation**	**Locality**	**GenBank number**
*E. parva* sp. nov.	Eucoparva1(♂)	Guizhou Prov., Dushan County, Mawei Town	PZ945055
*E. qinlingensis* sp. nov.	EucoQL(♂)	Shaanxi Prov., Mt. Qinling	PZ945043
*E. absens* sp. nov.	EucoSNJ01(♂)	Hubei Prov., Shennongjia Forestry District, Zhuheyuan	PZ945047
*E. westwoodi*	EucoWest1(♀)	Yunnan Prov., Dehong Prefecture, Yingjiang County	PZ945050
	EucoWest2(♂)		PZ945051
*E. linglong*	EucoLing1(♂)	Hainan Prov., Mt. Jianfengling	PZ945048
	EucoLing2(♂)		PZ945049
*E. dasytoides*	EucoDasyHN1(♂)	Hunan Prov., Changde City, Mt. Guniu	PZ945045
	EucoDasyHN2(♂)		PZ945046
	EucoDasyGZ(♂)	Guizhou Prov.	PZ945044
	TWN01	/	LC480880
	TWN02	/	LC480881
*E. yasumatsui*	EucuYasu1(♂)	Japan	PZ945053
	EucoYasu2(♂)		PZ945054
	EucoYasu3	/	LC480869
*E. tokarensis*	EucoToka1	/	LC480874
	EucoToka2	/	LC480875
*E. miyakoensis*	EucoMiya1	/	LC565397
	EucoMiya2	/	LC565398
*E. donanensis*	EucoDona1	/	LC480877
	EucoDona2	/	LC480878
**Outgroup**			
*Ergaula nepalensis*	ErgaNepa	/	NC_082502
*Diploptera punctata*	DiplPunc	/	MF49156

For phylogenetic analysis, the optimal partition scheme and substitution model were determined using PartitionFinder v. 2.1.1 (Lanfear et al. 2017) with default parameters. The maximum likelihood (ML) phylogenetic tree was reconstructed with ten independent likelihood searches performed in IQ-TREE v. 2.2.0 (Nguyen et al. 2015) to identify the best-supported topology. The result with the highest likelihood value was selected for presentation. Node and branch support was evaluated with 10,000 ultrafast bootstrap (UFBoot) replicates. In addition, the ‘-bnni’ option was used to mitigate severe model violations, and finally visualization was performed using FigTree v. 1.4.4 (http://tree.bio.ed.ac.uk/software/figtree/). Molecular species delimitation was performed using the ASAP method (Puillandre et al. 2021), based on pairwise K2P genetic distances with default parameters and a recursive split threshold of 0.01. The optimal species partition was determined by the minimum ASAP score, which integrates the probability of panmixia and the relative barcode gap width.

The GMYC method requires a fully resolved, strictly ultrametric bifurcating tree as input. To meet this requirement, a Bayesian inference tree was reconstructed in BEAST 1.8.2 using the best-fit nucleotide substitution model selected by PartitionFinder2. An uncorrelated lognormal relaxed-clock model was applied to accommodate rate variation among branches. The mean clock rate was estimated under a uniform prior distribution with a lower boundary of 0, an upper boundary of 1, and an initial value of 0.0001. A UPGMA tree was used as the starting tree, and a birth-death model was specified as the tree prior. Two independent Markov Chain Monte Carlo (MCMC) analyses were executed for 5 million generations each, with sampling every 500 generations. The resulting trees were then summarized using TreeAnnotator in the BEAST package to generate a maximum clade credibility tree, with the first 10% of sampled trees from each run discarded as burn-in. Convergence was assessed with effective sample size (ESS) values ≥ 200 in Tracer v. 1.6.0 (Drummond and Rambaut 2007).

The final ultrametric gene tree was submitted to the GMYC online server (https://species.h-its.org/gmyc/) for species delimitation using the single-threshold algorithm. The backend of this web server implements the original R version of the GMYC model (Fujisawa and Barraclough 2013). The delimitation results were evaluated using a likelihood-ratio test, which statistically compares the multiple-species clustering model with a single-species null model.

## Results

### Molecular analysis based on *COI*

**Figure 1.**
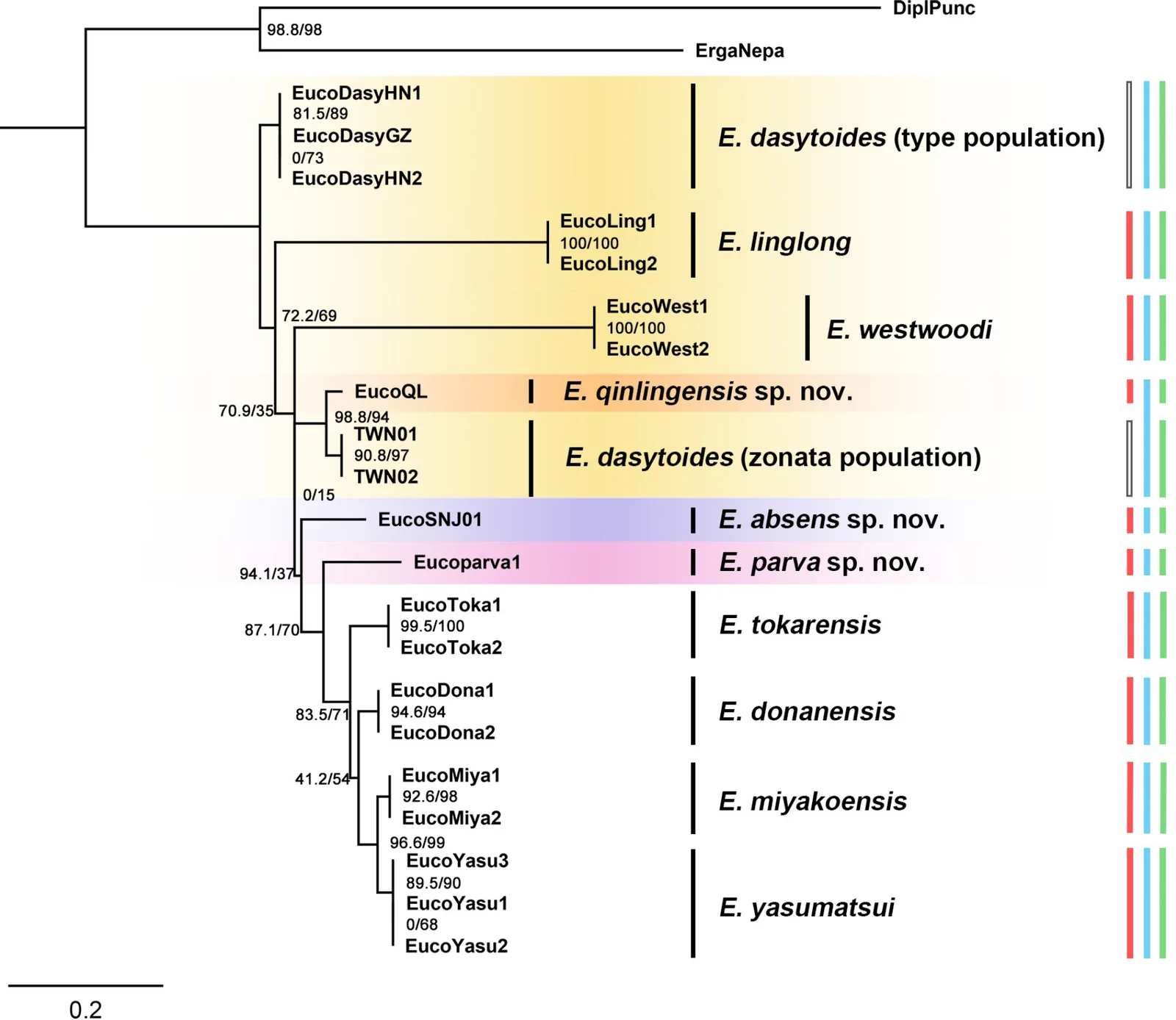
Phylogenetic relationships inferred from *COI* sequences using Maximum Likelihood. Numbers at nodes indicate bootstrap support values. Red bar: results of morphological delimitation; *E. dasytoides* in black frame. Blue bar: results of MOTUs in ASAP. Green bar: results of MOTUs in GMYC.

Maximum likelihood (ML) analysis of 23 *COI* sequences (Fig. 1) recovered all conspecific clades (including both males and females) as well supported monophyletic groups, except for *E. dasytoides*. The zonata population of *E. dasytoides* clustered with *E. qinlingensis* sp. nov. rather than with the type population.

The ASAP analysis identified ten molecular operational taxonomic units (MOTUs) (Fig. 1, blue), largely congruent with morphological identification results. However, incongruence between molecular and morphological delimitation was observed in *E. dasytoides*: the zonata and the type populations were recovered as two independent MOTUs, whereas the zonata lineage and *E. qinlingensis* sp. nov. were delimited as a single MOTU.

The GMYC analysis identified 11 MOTUs (Fig. 1, green), in contrast to the ASAP results, the zonata population and *E. qinlingensis* sp. nov. were resolved as two separate MOTUs.

Interspecific genetic distances ranged from 3.39% (between the zonata population of *E. dasytoides* and *E. qinlingensis* sp. nov.) to 22.21% (between *E. linglong* and *E. yasumatsui*). The maximum intraspecific *COI* genetic divergence of *E. dasytoides* reached 8.67%, occurring between the geographical sample from Guizhou Prov. and Taiwan Prov., respectively (Suppl. material 1).

### Key to *Eucorydia* species within China (based males)

**Table:** 

1	Antennae without meristal annuli (Fig. 5E)	***E. absens* sp. nov**.
–	Antennae with meristal annuli	**2**
2	Tegmina with three heart-shaped spots	***E. westwoodi* (Gerstaecker, 1861)**
–	Not as above	**3**
3	Apical part of tegmina with a small yellow spot on each side and a band of white pubescence between them	***E. linglong* Qiu, Che & Wang, 2017**
–	Not as above	**4**
4	Apical half of tegmina uniformly yellow	**5**
–	Apical half of tegmina not entirely yellow	**7**
5	Anal region of each tegmen with a metallic bluish-green spherical area, the two areas disconnected	***E. xizangensis* Woo & Feng, 1988**
–	Not as above	**6**
6	Boundary between metallic and yellow areas on tegmina nearly straight	***E. hilaris* (Kirby, 1903)**
–	Boundary between metallic and yellow areas on tegmina W-shaped	***E. splendida* Qiu, Che & Wang, 2017**
7	Base of tegmina with white or gray pubescence	**8**
–	Base of tegmina without pale pubescence	**11**
8	Pronotum with short yellowish white pubescence, posterior margin of supra-anal widely concave	***E. guilinensis* Qiu, Che & Wang, 2017**
–	Not as above	**9**
9	Body length (excluding tegmina) ≤ 14 mm (Fig. 2A, B)	***E. parva* sp. nov**.
–	Body length (excluding tegmina) > 14 mm	**10**
10	Last two sternites black, remaining sternites orange	***E. pilosa* Qiu, Che & Wang, 2017**
–	Last two sternites black; remaining sternites medially black and laterally orange	***E. tangi* Qiu, Che & Wang, 2017**
11	Tegmina slightly exceeding abdomen (< 4 mm); R2 elongate	***E. yunnanensis* Woo, Guo & Feng, 1986**
–	Tegmina far exceeding abdomen (> 6 mm); R2 rounded	**12**
12	Posterior margin of supra-anal plate nearly straight	***E. dasytoides* (Walker, 1868)**
–	Posterior margin of supra-anal plate deeply concave (Fig. 3F)	***E. qinlingensis* sp. nov**.

### Taxonomy

#### Genus Eucorydia

Taxon classificationAnimaliaBlattodeaCorydiidae

Hebard, 1929

7D328CF4-AED2-50D7-9AAA-9D41C5436212

Eucorydia Hebard, 1929: 96; Princis 1963: 81; Asahina 1971: 256.

##### Type species.

*Eucorydia westwoodi* (Gerstaecker, 1861).

#### Eucorydia parva

Taxon classificationAnimaliaBlattodeaCorydiidae

Cai & Che sp. nov.

FADD5C43-1E9D-54DA-9A23-ED6572C8FC7A

https://zoobank.org/313C6A3B-2180-49AB-93E1-D8547C9BF9A1

Fig. 2

##### Type material.

***Holotype***: China • male; Guizhou Prov., Dushan County, Mawei Town; 9 June 2019; Lu Qiu leg; SWU-BB-CC-025001.

##### Diagnosis.

The new species resembles the Hainan population of *E. dasytoides* but is distinctly smaller (overall length 14.00 mm vs 18.60–22.30 mm in *E. dasytoides*; Qiu et al. 2017). The orange-yellow transverse band on the tegmina is markedly narrower in the new species. Additionally, the last four abdominal segments are entirely black in *E. parva* sp. nov., whereas in the Hainan population of *E. dasytoides* only the last three segments are black (Qiu et al. 2017: fig. 4G), with the lateral margins of segments 4 and 5 black (Qiu et al. 2017: fig. 2A, B).

**Figure 2.**
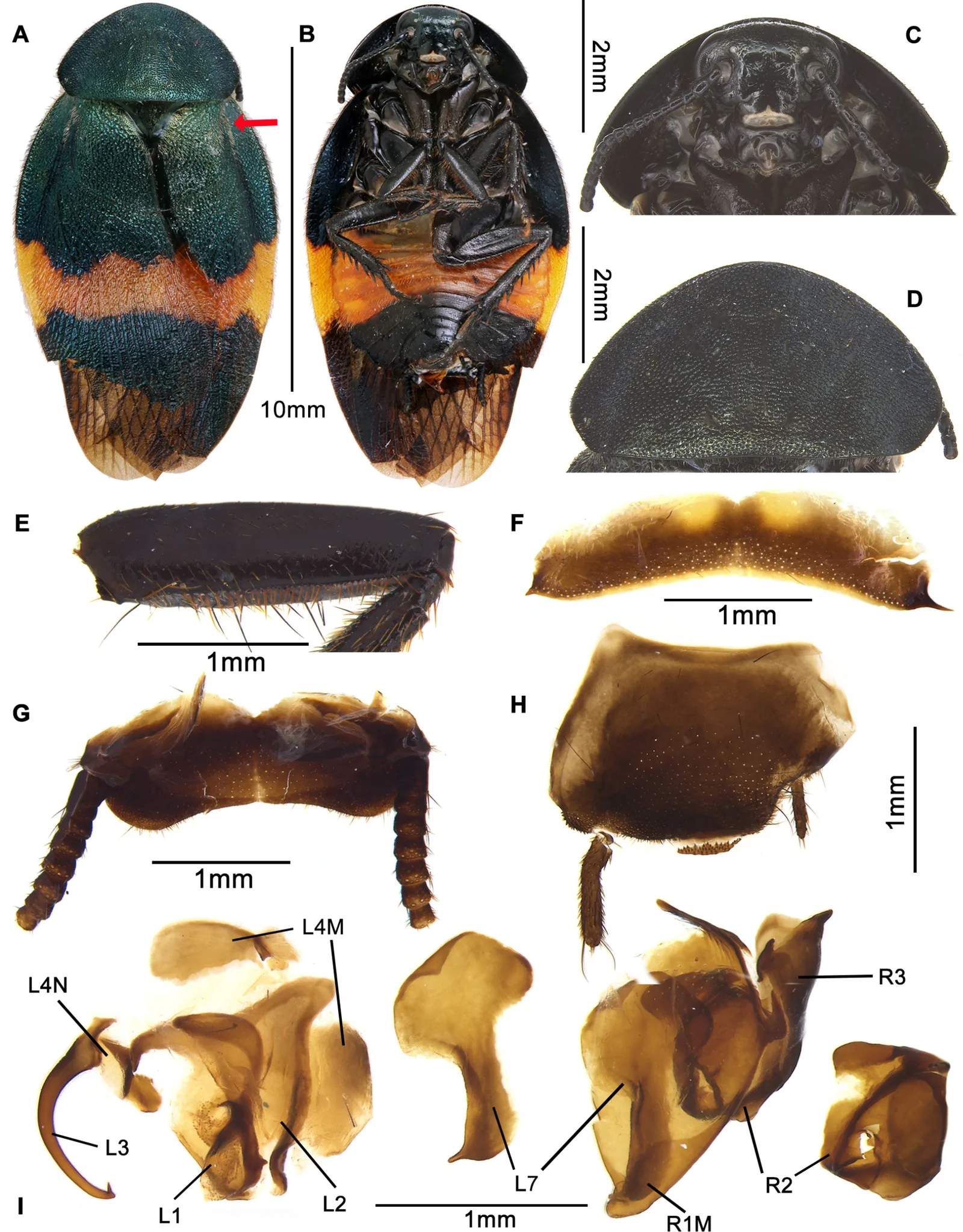
*Eucorydia parva* Cai & Che, sp. nov. **A**. Male holotype, dorsal view, red arrow points to the area with white setae; **B**. Male holotype, ventral view; **C**. Head, ventral view; **D**. Pronotum, dorsal view; **E**. Front femur **F**. T8, dorsal view; **G**. Supra-anal plate, ventral view; **H**. Subgenital plate, dorsal view; **I**. Genitalia, dorsal view.

##### Description.

**Holotype** (male). ***Measurements*** (mm). Overall length: 14.00; body length: 11.00; body width (excluding tegmina): 6.37; tegmen length × width: 12.30 × 5.00; pronotum length × width: 2.10 × 4.10.

***Coloration***. Pronotum metallic greenish blue, with black setae. Tegmina with basal half metallic greenish blue, a transverse orange-yellow median band, and apical half nearly black; basal pubescence white, hind wings brown. Head metallic dark blue. Eyes black, ocelli, antennal sockets, and ante-clypeus white. Antennae (except the white apical portion), labial palpi, and maxillary palpi dark brown. Legs dark brownish black, covered with brown pubescence. Leg spines dark brown with reddish brown tips. Last four abdominal sternites blackish brown, remaining sternites yellow.

***Head***. Slightly rounded, subtriangular. Eyes widely separated, ocelli small and rounded. Interocular space wider than the distance between antennal sockets, and the latter wider than the distance between ocelli. Two shallow depressions present between the antennal sockets. Labrum with a round median impression (Fig. 2C). ***Pronotum***. Transverse, densely nodulose and setose, widest near the posterior margin, median area with dark stripes (Fig. 2D). ***Tegmina and hind wings***. Basal region of tegmina with a patch of white pubescence (Fig. 2A). Sc swellings present. ***Legs***. Slender. Front femur Type C1 (Fig. 2E). Tibiae with multiple big spines, including its apical part. Pulvilli and arolia present. Tarsal claws symmetrical, unspecialized. ***Abdomen***. T8 specialized and asymmetrical; posterolateral corners produced and spinose (Fig. 2F). Supra-anal plate transverse, with posterior margin rounded and broadly concave, posterolateral corners rounded. Paraprocts basally bifurcate, extending outward, irregularly narrow, lamellate. Cerci long and slender (Fig. 2G). Subgenital plate asymmetrical; posterior margin nearly straight; left margin concave upward, bearing a left stylus distinctly smaller than the right; right stylus angled at proximal third (Fig. 2H). ***Genitalia***. Strongly sclerotized. L2 irregularly plate-like; left portion extended into a slender, curved, arm-like process, right portion with a rounded, blunt protrusion. L3 (genital hook) curved, with apex sharp. Basal portion of L7 extended and convex, apical part distinctly narrowed. R2 slightly rounded (Fig. 2I).

**Female**. Unknown.

**Nymph**. Unknown.

**Ootheca**. Unknown.

##### Natural history.

The male holotype was found freshly dead on a roadside at the edge of a town surrounded by karst landscapes.

##### Etymology.

The specific epithet is derived from the Latin adjective *parvus*, meaning “small*”*, and refers to the relatively small body size of this species compared with its congeners. The name is feminine in gender.

##### Remarks.

Interspecific genetic distances between the new species and its sequenced congeners range from 10.85–19.77%. The shapes of L7 and R2 are similar to those of *E. dasytoides* (Qiu et al. 2017), but the new species can be easily distinguished by the presence of white pubescence at the base of the tegmina and its significantly smaller body size.

#### Eucorydia qinlingensis

Taxon classificationAnimaliaBlattodeaCorydiidae

Cai & Che sp. nov.

34F8C8EA-0823-5914-A778-3B41035FF9B5

https://zoobank.org/3B4602B0-AD48-40C0-98C8-FFC8792EEF78

Figs 3, 4

##### Type material.

***Holotype***: China • male; Shaanxi Prov., Mt. Qinling; July 2022; collector unknown; SWU-BB-CC-026001. ***Paratype***: China • 2 male & 2 females; same collection data as holotype; SWU-BB-CC-026002 to SWU-BB-CC-026005.

##### Diagnosis.

**Figure 3.**
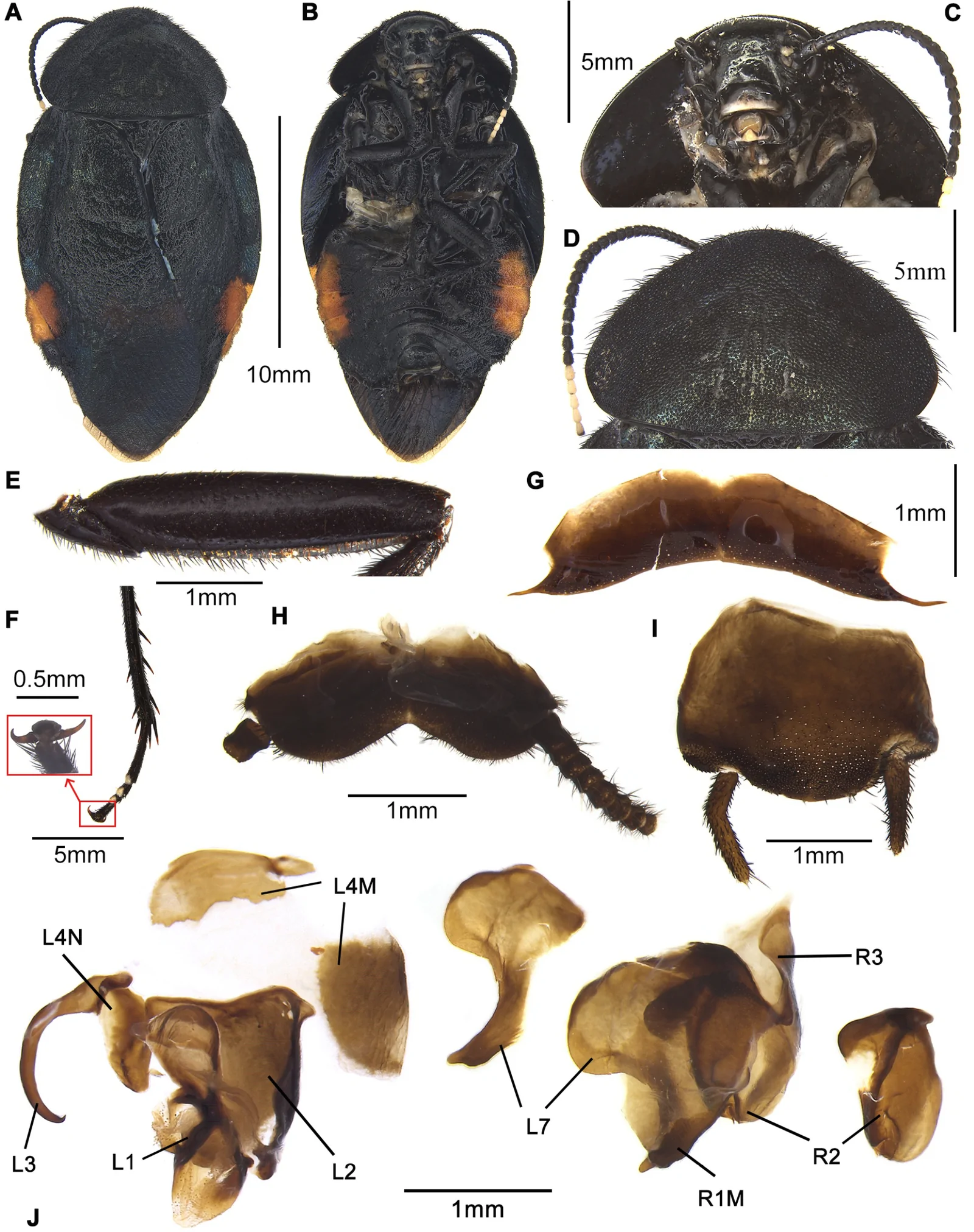
*Eucorydia qinlingensis* Cai & Che, sp. nov. **A**. Male holotype, dorsal view; **B**. Male holotype, ventral view; **C**. Head, ventral view; **D**. Pronotum, dorsal view; **E**. Front femur; **F**. Hind tarsi **G**. T8, dorsal view; **H**. Supra-anal plate, ventral view; **I**. Subgenital plate, dorsal view; **J**. Genitalia, dorsal view.

The new species is externally similar to the *purpuralis* population of *E. dasytoides* (Zhejiang Prov., Mt. Tianmushan; Qiu et al. 2017: fig. 5F, G), but can be readily distinguished by the shape of the male supra-anal plate: the posterior margin is shallowly emarginated in *E. dasytoides*, whereas deeply concave in the new species (Fig. 3H).

##### Description.

**Holotype** (male). ***Measurements*** (mm). Overall length: 19.24; body length: 16.04; body width (excluding tegmina): 8.20; tegmen length × width: 13.74 × 5.53; pronotum length × width: 7.97 × 4.62.

***Coloration***. Pronotum metallic bluish-green, covered with black setae. Tegmina with three orange-yellow spots, the basal part metallic bluish-green, and the apical part dark brown. Hind wings uniformly brown. Head metallic dark blue. Eyes black, ocelli and antennal sockets white. Hind-clypeus black, Ante-clypeus white. Middle part of labrum white, dark brown on the remaining parts. Antennae (excluding the whitish apical segments), labial palpi and maxillary palpi black. Legs black, covered with black pubescence, spines on legs dark blackish-brown with reddish-brown apices. Pulvilli white, arolia nearly black. Sternites black, with orange-yellow lateral stripes on sternites III–V.

***Head***. Drop-shaped, slightly extending beyond the pronotum, frons flat. Interocular space relatively wide, distance between ocelli nearly equal to that between antennal sockets, both smaller than interocular space. Ocelli small. Middle of labrum prominently raised into a mound-like shape, with a distinct small tongue-shaped protrusion at the center of the lower margin (Fig. 3C). ***Pronotum***. Triangular, covered with short pubescence (Fig. 3D). ***Tegmina and hind wings***. Tegmina with Sc swellings present, with orange markings on the lateral margin and two obscure orange markings medially, hind wings without markings. ***Legs***. Slender. Front femur Type C1. Tibiae with multiple large spines, pulvilli and arolia present. Tarsal claws unspecialized (Fig. 3E, F). ***Abdomen***. Covered with short brown setae (Fig. 3B). T8 specialized and slightly asymmetrical; posterolateral corners spinose and produced, with the right projection longer than the left (Fig. 3G). Supra-anal plate transverse, posterior margin broadly arcuate with a deep median concavity, lateral corners rounded and protruding. Paraprocts basally bifurcate, extending outward, irregularly narrow, lamellate. Cerci slender (Fig. 3H). Subgenital plate covered with short black setae, anterior margin asymmetrical, posterior margin produced, right stylus angled at proximal third and slightly larger than left (Fig. 3I). ***Genitalia***. Highly sclerotized. L2 irregularly plate-like; left portion extending into a slender, curved arm-like process, right portion with an acute protrusion. L3 arcuate with acute apex. L7 subrounded in basal half, evenly elongated in distal half, with a bud-like apex. R2 slightly elongated, basal right with a corniform protrusion (Fig. 3J).

**Male (paratypes)**. Generally matching holotype. Overall length: 19.24; body length: 16.04; body width (excluding tegmina): 8.63; tegmen length × width: 15.33 × 6.13; pronotum length × width: 7.97 × 4.62.

**Female** (paratypes). ***Measurements* (mm)**. Overall length: 13.38–13.45; body length: 13.02–13.25; body width (excluding tegmina): 8.32–8.46; tegmen length × width: 10.15–10.16 × 4.71–5.15; pronotum length × width: 6.69–6.94 × 3.40–4.36.

***Coloration***. Similar to male holotype, except orange-yellow tegminal markings indistinct medially and head almost entirely black.

**Figure 4.**
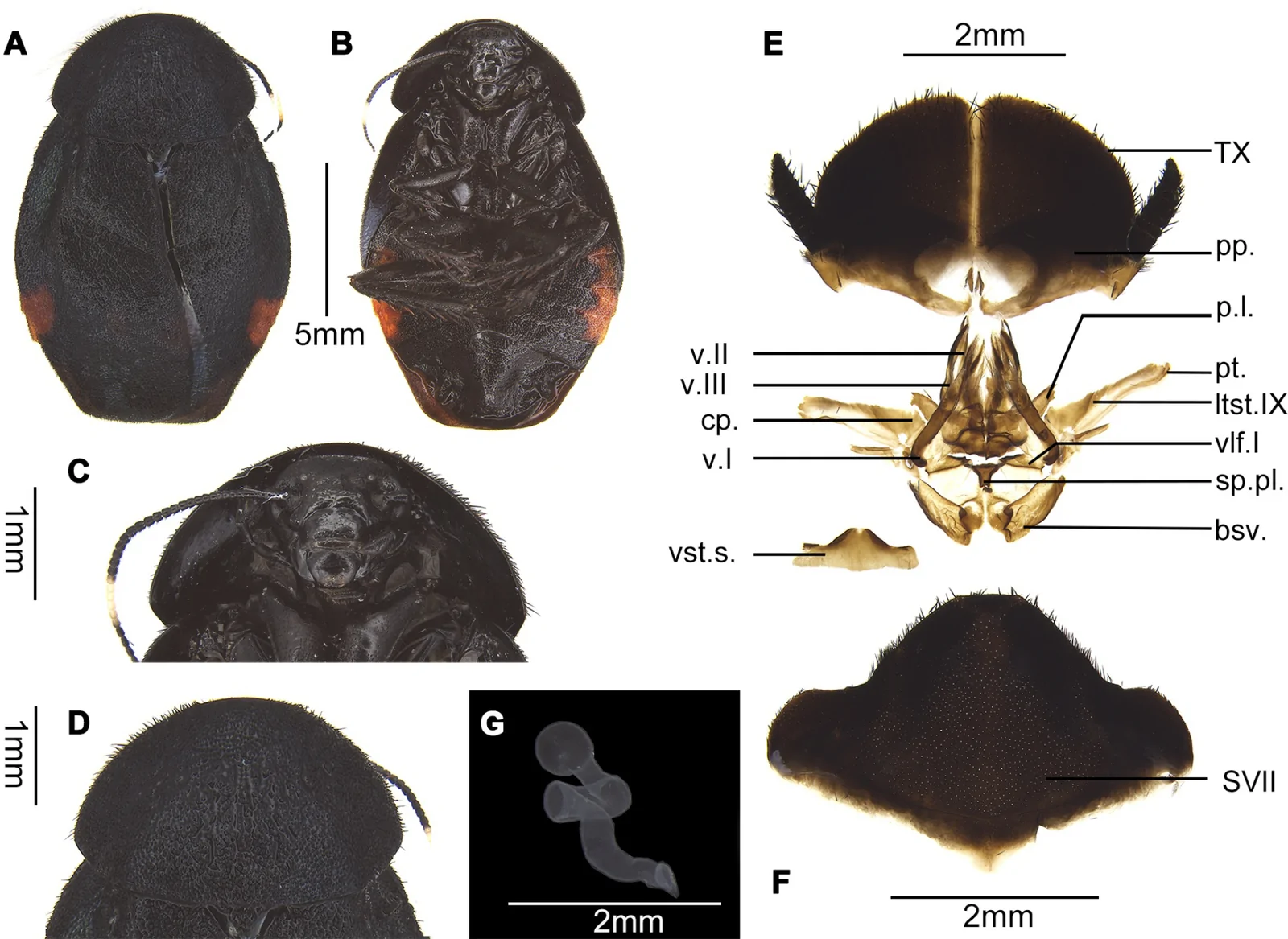
*Eucorydia qinlingensis* Cai & Che, sp. nov. **A**. Female paratype, dorsal view; **B**. Female paratype, ventral view; **C**. Head, ventral view; **D**. Pronotum, dorsal view; **E**. Genitalia and supra-anal plate; **F**. Subgenital plate; **G**. Spermatheca.

***Head***. Vertex with pubescence, maxillary and labial palpi pubescent. Frons convex, labrum nearly trapeziform (Fig. 4C). ***Pronotum***. Subelliptical and pubescent, widest near the posterior margin (Fig. 4D). ***Tegmina and hind wings***. The posterior margin of tegmina nearly level with the end of abdomen (Fig. 4A). ***Legs***. Slender, pulvilli and arolia present (Fig. 4B). ***Abdomen***. S3-S5 with yellow lateral maculae; tergites unspecialized. Supra-anal plate dark blackish-brown, posterior margin slightly concave medially, surface setose (Fig. 4E). Subgenital plate without valvar; apical margin arcuately produced, surface setose (Fig. 4F). ***Genitalia***. Paraprocts with long marginal setae; middle of the inner margin extending inward to form a leaf-like protrusion; cerci short, segmented, densely setose. Paratergites narrow and band-shaped. Crosspiece relatively large. Posterior lobes of valvifer II distinct, apex irregular. Ovipositors large and slender, surface smooth. Spermathecal plate highly sclerotized, medially constricted, trapezoidal. Basivalvula broad, with two spherical protrusions medially. Vestibular sclerite highly sclerotized, hillock-shaped (Fig. 4E). Spermathecal duct short, spermatheca tubular with expanded and spiraled apex (Fig. 4G).

**Nymph**. Unknown.

**Ootheca**. Unknown.

##### Etymology.

The specific epithet *qinlingensis* refers to the Mt. Qinling, the type locality of this species. The name is feminine in gender.

##### Remarks.

This species differs morphologically from the zonata population of *E. dasytoides* by the following characters: 1) the orange band on the tegmina is markedly wider in the latter (Qiu et al. 2017: fig. 4A–D); 2) the posterior margin of the male supra-anal plate is relatively flat in the latter (Qiu et al. 2017: fig. 6B), whereas it is distinctly concave in the new species; and 3) the two taxa are geographically isolated, with a straight-line distance approximately 1700 km between the Mt. Qinling and Mt. Lalashan (Taiwan Prov.). Despite the relatively low genetic distance (3.39%), the combined evidence from morphological differences, genitalic characters and geographic isolation support their recognition as distinct species.

#### Eucorydia absens

Taxon classificationAnimaliaBlattodeaCorydiidae

Cai & Che sp. nov.

74E94384-BA06-53D3-9752-95B3A00921DF

https://zoobank.org/C12DA707-3D74-4969-8602-F4DFE04000D7

Fig. 5

##### Type material.

***Holotype***: China • male; Hubei Prov., Shennongjia Forestry District, Zhuheyuan; June 2023; Chun-Hong Wang leg; SWU-BB-CC-027001.

##### Diagnosis.

**Figure 5.**
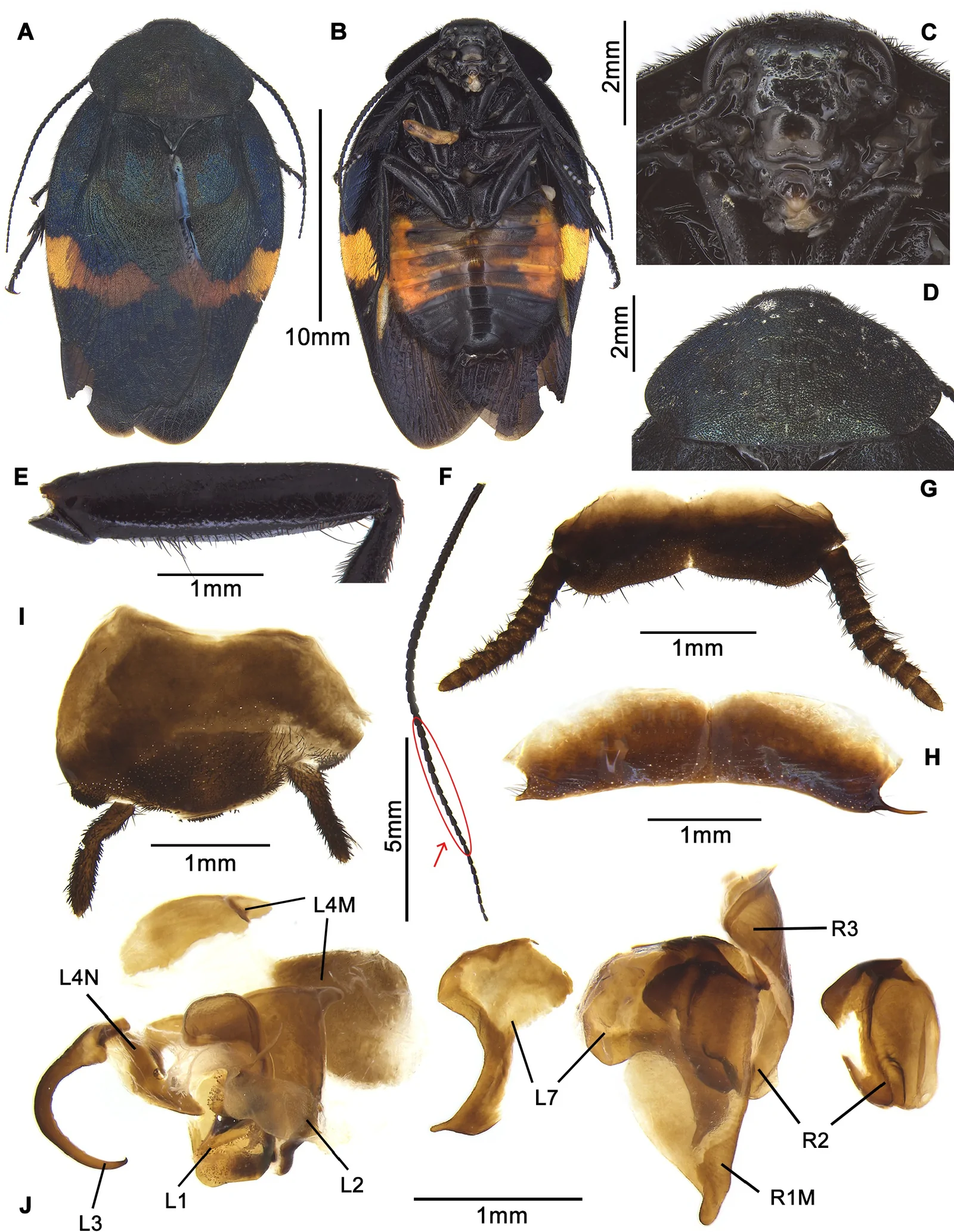
*Eucorydia absens* Cai & Che, sp. nov. **A**. Male holotype, dorsal view; **B**. Male holotype, ventral view; **C**. Head, ventral view; **D**. Pronotum, dorsal view; **E**. Front femur; **F**. Antenna, showing absence of meristal annuli; **G**. Supra-anal plate, ventral view **H**. T8, dorsal view; **I**. Subgenital plate, dorsal view; **J**. Genitalia, dorsal view.

This species can be readily distinguished from other congeners by its entirely black antennae lacking meristal annuli (Fig. 5F). It otherwise resembles the zonata population of *E. dasytoides*, but differs in the following characters: tegminal markings: the orange-yellow markings possess a median fissure, and the curvature of L3 is distinctly stronger than in *E. dasytoides*.

##### Description.

**Holotype** (male). ***Measurements*** (mm). Overall length: 22.32; body length: 18.01; body width (excluding tegmina): 8.62; tegmen length × width: 16.32 × 6.88; pronotum length × width: 7.96 × 3.96.

***Coloration***. Head, eyes and antennae black, ocelli white. Ante-clypeus with a white band-shaped marking; labrum blackish-brown, with only the apex of the median protrusion white. Pronotum metallic bluish-green. Tegminal overall metallic bluish-green, middle part with a yellow band. Legs dark brownish-black, covered with brown pubescence, spines dark brown with reddish-brown apices. Eversible glands, pulvilli, and arolia white. The last four sternites black, remaining sternites orange-yellow and median with symmetrical black marking.

***Body***. Slightly broad (Fig. 5A-B). ***Head***. Inverted triangular, almost entirely covered by pronotum. Frons flat, with two small medial depressions. Interocular space relatively wide, distance between ocelli slightly smaller than that between antennal sockets, both narrower than interocular space. Ocelli small, rounded (Fig. 5C). ***Pronotum***. Transverse, widest near the posterior margin, densely covered with short setae, with indistinct dark longitudinal stripes medially (Fig. 5D). ***Tegmina and hind wings***. Tegmina with Sc swellings present. Hind wings hyaline, apex brown, anterior margin of the RA area with an elongate yellow spot, venation brown (Fig. 5A). ***Legs***. Slender. Front femur Type C1 (Fig. 5E). Tibiae with multiple large spines, pulvilli and arolia present. Tarsal claws symmetrical, unspecialized (Fig. 5B). ***Abdomen***. Setose, eversible glands distinct. Posterior margin of the supra-anal plate constricted medially, forming a clear notch, lateral posterior angles rounded. Paraprocts basally bifurcate, extending outward, irregularly narrow, and lamellate; median sclerites lost during dissection (Fig. 5G). T8 specialized and asymmetrical, posterolateral angles spinose-produced and pubescent (Fig. 5H). Subgenital plate asymmetrical and densely setose; styli subequal in length, each angulate to varying degrees at proximal third (Fig. 5I). ***Genitalia***. Highly sclerotized. L2 plate-like, left portion extending into a slender, curved arm-like process, right portion with a corniform protrusion. L3 strongly curved, semicircular, with a small hook at the apex. L7 with base rounded, medially elongated, apex bud-shaped. R2 nearly elongate-elliptical (Fig. 5J).

**Female**. Unknown.

**Nymph**. Unknown.

**Ootheca**. Unknown.

##### Etymology.

The specific epithet *absens* is a Latin adjective, meaning absent, and refers to the absence of meristal annuli. The name is feminine in gender.

##### Remarks.

The species resembles the zonata population of *E. dasytoides*, but differs in the medially interrupted tegminal markings, a more strongly curved L3, and genetic distances of 9.35–18.93% from its congeners. These combined morphological and molecular differences support its recognition as a distinct species.

#### Eucorydia westwoodi

Taxon classificationAnimaliaBlattodeaCorydiidae

(Gerstaecker, 1861)

B145A53B-DEA3-5A89-A256-9F1AE75CE660

Fig. 6

Corydia westwoodi Gerstaecker, 1861: 114.
Eucorydia westwoodi : Hebard 1929: 96; Qiu et al. 2017: 48; Qiu et al. 2019: 6.

##### New material examined.

China • 1 female; Yunnan Prov., Dehong Jingpo Autonomous Prefecture, Yingjiang County, Shangbangwa Village; 2021; Jia-Zhi Zhang leg; SWU-BB-CC-021028 • 1 female; Yunnan Prov., purchased from the cockroach pet market; SWU-BB-CC-021023.

**Figure 6.**
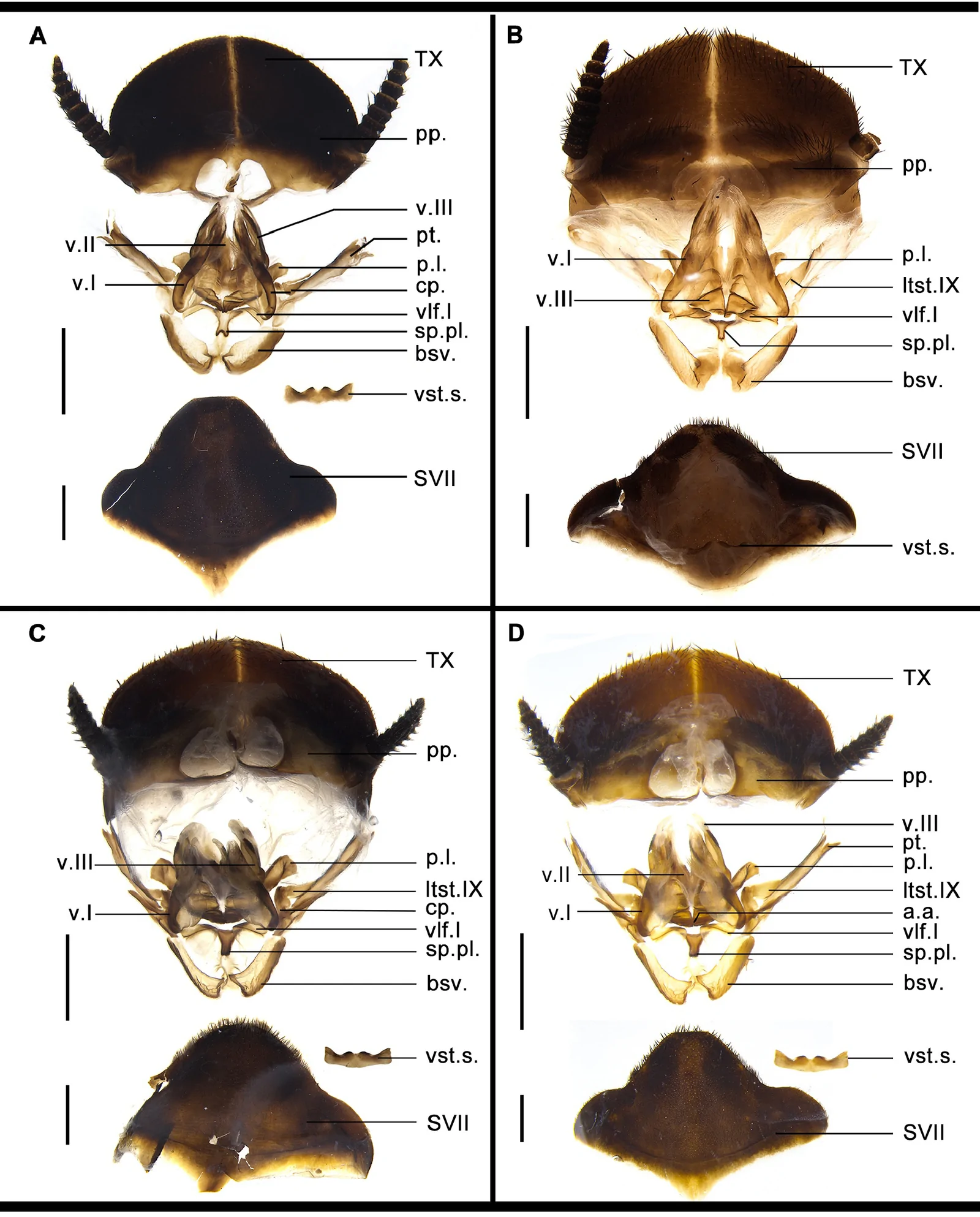
Female genitalia of four *Eucorydia* species (supra-anal plate and subgenital plate included). **A**. *Eucorydia westwoodi* (Gerstaecker, 1861); **B**. *Eucorydia dasytoides* (Walker, 1868); **C**. *Eucorydia linglong* Qiu, Che & Wang, 2017; **D**. *Eucorydia guilingensis* Qiu, Che & Wang, 2017. Scale bars: 1 mm.

##### Female genitalia.

Supra-anal plate dark yellowish brown, posterior margin evenly rounded, surface setose. Paraprocts with long setae along the outer margin, inner margin medially curved, with two median sclerites present; cerci segmented, densely setose. Paratergites narrow and strap-shaped, with a bifurcated apex. Crosspiece relatively small, connected to the dorsal plate. Posterior lobes of valvifer II distinct, with a robust apex. First valvule large and slender, surface smooth, fused with the spermathecal plate. Spermathecal plate strongly sclerotized, medially constricted, curved downward, and apically bearing two small angular processes. Lateral lobe of basivalvula broad, roughly triangular, with differently curved inner and outer margins. Spermathecal duct short. Spermatheca tubular, apex swollen. Vestibular sclerite strongly sclerotized, with two median projections and a distinct depression. Subgenital plate without valvar; distal margin rounded and produced, and surface setose.

#### Eucorydia dasytoides

Taxon classificationAnimaliaBlattodeaCorydiidae

(Walker, 1868)

F0BDBCB0-D6E9-58A7-99A8-AA663EA4E265

Fig. 6

Euthyrrhapha dasytoides Walker, 1868: 191.
Eucorydia dasytoides : Hebard 1929: 98; Qiu et al. 2017: 24.
Corydia tonkinensis Kirby, 1903: 405. Synonymized by Kirby (1904).
Eucorydia tonkinensis : Hebard 1929: 97.
Corydia purpuralis Kirby, 1903: 405. Synonymized by Qiu et al. (2017).
Eucorydia purpuralis : Hebard 1929: 98.
Corydia zonata Shiraki, 1907: 110. Synonymized by Princis (1963).
Eucorydia paucipilosa Woo et al., 1986: 156. Synonymized by Qiu et al. (2017).

##### New material examined.

China • 1 female; Hunan Prov., Yongzhou City, Ningyuan County, Mt. Jiuyi Sanfenshi Scenic Spot; 5 June 2021; Jing Zhu leg; SWU-BB-CC-022066 • 1 female; Hunan Prov., Huaihua City, Yuanling County, collector unknown; SWU-BB-CC-022067.

##### Material examined for female genitalia.

China • 1 female, Hunan Prov., Huaihua City, Yuanling County, data unknown; SWU-BB-CC-022007 • 1 female, Hunan Prov., Yueyang City, 6 Jun, Liu & Wang (only surnames) leg; SWU-BB-CC-022008 • 1 female, Guizhou Prov., Tongren City, Jiangkou County, Mt. Fanjingshan, 12 July 1988, Shu-Yong Wang leg; SWU-BB-CC-022009 • 1 female, Guizhou Prov., Zhenyuan County, Tiexi, 25 May 2016, Yong-Shan Guo & Shu-Lin Yang leg; SWU-BB-CC-022010 • 1 female, Guizhou Prov., Yanhe County, Daheba, 450–700 m, 5–12 Jun 2007, Qiong-Zhang Song leg; SWU-BB-CC-022016.

##### Female genitalia.

Supra-anal plate yellowish brown, surface setose, posterior margin rounded. Paraprocts medially depressed. Two median sclerites present. Paratergites band-shaped. Laterosternite IX lamellar, attached to the crosspiece and paratergites. Posterior lobes of valvifer II distinct, apical portion slightly curved. First valve elongate, basal portion of third valve broad. First valvifer lamellate, with an oblique fold. Spermathecal plate with basal half subtrapezoidal and apical half subrectangular. Basivalvula narrow, symmetrical; lobes bulbous and expanded proximally. Spermathecal duct short. Spermatheca elongate, tubular, with both ends expanded (Liu et al. 2023: fig. S26H). Vestibular sclerite with margins poorly defined, median region bearing a large protrusion. Subgenital plate without valvar; posterior margin produced, and surface densely setose.

#### Eucorydia linglong

Taxon classificationAnimaliaBlattodeaCorydiidae

Qiu, Che & Wang, 2017

BBB5F9FD-28DB-54A2-9626-531557CA90C4

Fig. 6

Eucorydia linglong Qiu et al., 2017: 31.

##### Type locality.

Hainan Prov., Ledong County, top of Mt. Jianfengling.

##### Material examined for female genitalia.

China • 1 female (Paratype); Guangxi Zhuang Autonomous Region, Chongzuo City, Daxin County, Shuolong Town; 20 May 2016; Yi-Zhou Liu leg; SWU-BB-CC-023002.

##### Female genitalia.

Supra-anal plate dark yellowish brown, covered with long setae, posterior margin roundly produced. Paraprocts medially depressed, with a slender, curved hook-like process. Two median sclerites present. Paratergites narrow and band-shaped, laterosternite IX attached to crosspiece and paratergites. Posterior lobes of valvifer II robust, apical portion slightly expanded with well-defined margins. Valves short, basal part of third valvule broad. First valvifer lamellate, attached to the spermathecal plate. Spermathecal plate with basal half sub-trapezoidal and the apical half sub-rectangular. Basivalvula narrow, with lobes unequal in size (left lobe narrower, right lobe slightly wider). Spermathecal duct short. Spermatheca elongate, tubular, with both ends expanded (Liu et al. 2023: fig. 4(9)). Vestibular sclerite with well-defined, undulate anterior and posterior margins, median region with two protrusions. Subgenital plate without valvar; posterior margin produced, and surface densely setose.

#### Eucorydia guilingensis

Taxon classificationAnimaliaBlattodeaCorydiidae

Qiu, Che & Wang, 2017

F4D6948D-D17B-58CD-9831-FBD4EF194CA7

Fig. 6

Eucorydia guilingensis Qiu et al., 2017: 40.

##### Type locality.

Guangxi Zhuang Autonomous Region, Guilin City, Yanshan, 200 m.

##### New material examined.

China • 1 female, 3 nymphs & 8 oothecae; Guangxi Zhuang Autonomous Region, Guilin City, around Ludiyan; 21 Aug. 2020; Lu Qiu leg; SWU-BB-CC-024002 to SWU-BB-CC-024005.

##### Remarks.

The female genitalia of this species are very similar to that of *E. linglong*, but the apices of the posterior lobes of valvifer II are less robust. Therefore, they are not redescribed here.

### Notes on female genitalia of *Eucorydia*

We provide the first detailed comparative analysis of female genitalia in *Eucorydia*. Comparative analysis reveals consistently high sclerotization in four key structures across all examined species: spermathecal plate, basivalvula, vestibular sclerite, and laterosternite IX, which also exhibits species-specific variation, supporting their taxonomic value. The sclerotized portions of the basivalvular lobe are relatively narrow and are connected to the spermathecal plate by a membranous structure (Figs 4E, 6). The crosspiece typically articulates with the first valvifer and laterosternite IX, extending toward the posterior lobe of valvifer II (Figs 4E, 6), which exhibits interspecific variation in both shape and size. Interspecific differences are also evident in other characters: the first valvifer is folded in *E. dasytoides* but smooth in the other three species (Fig. 6A); the spermathecal plate bears two small apical horns in *E. westwoodi* but is nearly trapezoidal in the remaining species (Fig. 6B); and the basal end of the basivalvular lobe is acute in most species, but distinctly bulbous in *E. dasytoides* (Fig. 6A).

## Discussion

In this study, the minimum interspecific *COI* genetic distance was found to be 3.39%, observed between the zonata population of *E. dasytoides* and *E. qinlingensis* sp. nov. This value falls well below the maximum intraspecific variation of 8.67%, which was detected between the geographically separated samples from Guizhou and Taiwan Provinces, thereby resulting in an overlap in the DNA barcoding gap. Consequently, the distance-based ASAP analysis fails to delimit the two lineages as separate MOTUs. Nevertheless, morphological evidence, particularly the shape of the posterior margin of the male supra-anal plate, combined with results from the tree-based GMYC analysis, supports their recognition as distinct species.

Compared with related corydiid cockroaches, the minimum interspecific distance detected in *Eucorydia* is relatively low. For example, the reported minimum interspecific *COI* distances are 9.8% in *Eupolyphaga* and 5.80% in *Pseudoeupolyphaga* (Han et al. 2022; Ren et al. 2025). However, an overlap between intra- and interspecific genetic distances has also been reported in these genera, with maximum intraspecific distances reaching 9.54% in *Eupolyphaga* and 6.90% in *Pseudoeupolyphaga* (Han et al. 2022; Ren et al. 2025). These comparisons suggest that the absence of a clear DNA barcoding gap may not be unique to *Eucorydia*, but may occur more broadly among related corydiid taxa.

These results highlight the limitations of relying exclusively on *COI* sequences for species delimitation in *Eucorydia* and related genera, as single mitochondrial markers may be affected by incomplete lineage sorting, introgression, and recent divergence (Funk and Omland 2003; Dupuis et al. 2012). Although external morphology and color pattern are useful for preliminary identification, they may be insufficient when closely related species show limited mitochondrial divergence or when geographically structured populations exhibit high intraspecific variation (Will et al. 2005). Therefore, future studies should integrate broader geographic sampling, quantitative morphometric analyses, multi-locus nuclear markers, and genomic-scale data to clarify species boundaries and evaluate cryptic diversity in corydiid cockroaches.

## Supplementary Material

XML Treatment for Eucorydia

XML Treatment for Eucorydia parva

XML Treatment for Eucorydia qinlingensis

XML Treatment for Eucorydia absens

XML Treatment for Eucorydia westwoodi

XML Treatment for Eucorydia dasytoides

XML Treatment for Eucorydia linglong

XML Treatment for Eucorydia guilingensis

## Additional information

### Artificial Intelligence (AI) use

The authors accept full responsibility for the content of the manuscript, including the disclosure of any use of AI.

No AI tools were used in the preparation of this manuscript.

### Author ORCIDs

T.-T. Cai https://orcid.org/0009-0007-7796-2514

W. Han https://orcid.org/0000-0002-7243-1657

Z.-Q. Wang https://orcid.org/0000-0001-9413-1105

Y.-L. Che https://orcid.org/0000-0003-3214-9494

### Data availability

All of the data that support the findings of this study are available in the main text or Supplementary Information.

## Supplementary materials

Supplementary material 1 Supplementary information https://doi.org/10.3897/zookeys.1293.199475.suppl1 Data type xls Explanation note Genetic divergence of distances calculated by K2P model method using cytochrome c oxidase subunit I (COI) gene sequences in MEGA. https://binary.pensoft.net/file/1792781This dataset is made available under the Open Database License (http://opendatacommons.org/licenses/odbl/1.0/). The Open Database License (ODbL) is a license agreement intended to allow users to freely share, modify, and use this Dataset while maintaining this same freedom for others, provided that the original source and author(s) are credited. Ting-Ting Cai, Wei Han, Zong-Qing Wang, Yan-Li Che
